# Role of store-operated Ca2+ entry in cardiovascular disease

**DOI:** 10.1186/s12964-022-00829-z

**Published:** 2022-03-18

**Authors:** Ting Lu, Yihua Zhang, Yong Su, Dayan Zhou, Qiang Xu

**Affiliations:** Department of Cardiology, Chongqing Fifth People’s Hospital, No. 24 Renji Road, Chongqing, 400000 China

**Keywords:** Stromal interaction molecule 1, Ca2+ release-activated Ca2+ channel protein 1, Store-operated channels, Transient receptor potential ion channels, Cardiovascular disease

## Abstract

Store-operated channels (SOCs) are highly selective Ca2+ channels that mediate Ca2+ influx in non-excitable and excitable (i.e., skeletal and cardiac muscle) cells. These channels are triggered by Ca2+ depletion of the endoplasmic reticulum and sarcoplasmic reticulum, independently of inositol 1,4,5-trisphosphate (InsP3), which is involved in cell growth, differentiation, and gene transcription. When the Ca2+ store is depleted, stromal interaction molecule1 (STIM1) as Ca2+ sensor redistributes into discrete puncta near the plasma membrane and activates the protein Ca2+ release activated Ca2+ channel protein 1 (Orai1). Accumulating evidence suggests that SOC is associated with several physiological roles in endothelial dysfunction and vascular smooth muscle proliferation that contribute to the progression of cardiovascular disease. This review mainly elaborates on the contribution of SOC in the vasculature (endothelial cells and vascular smooth muscle cells). We will further retrospect the literature implicating a critical role for these proteins in cardiovascular disease.

**Graphical Abstract:**

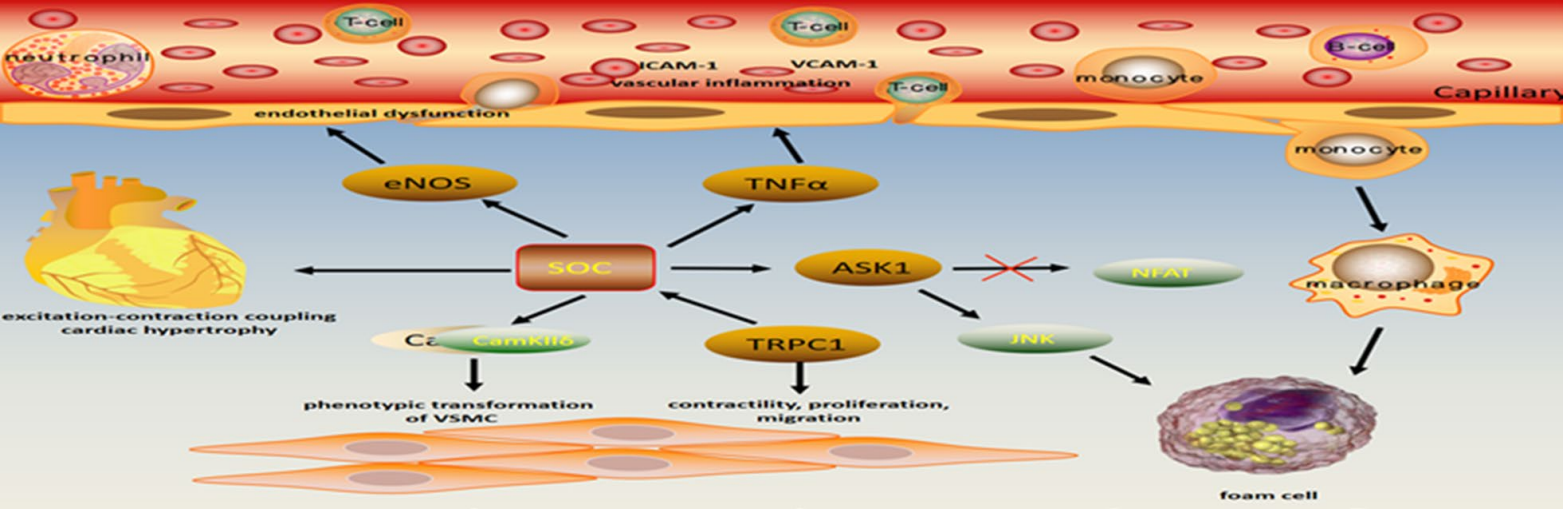

**Video Abstract**

**Supplementary Information:**

The online version contains supplementary material available at 10.1186/s12964-022-00829-z.

## Background

Calcium ions (Ca2 +) are second messengers and are widely involved in various physiological processes such as cell proliferation, muscle contraction, and enzyme regulation. The fluctuation of internal Ca2+ ions in response to receptor stimulation is usually achieved by releasing Ca2+ ions from intracellular Ca2+ ion stores or influx across the plasma membrane (PM) via Ca2+ ion-permeable channels and emerged as the Ca2+ release-activated Ca^2+^ channels (CRACs). CRAC is considered as prototypic SOC. Store-operated Ca2+ entry (SOCE) is a ubiquitous Ca2+ influx mechanism, expressed in non-excitable and excitable cells, triggered by the depletion of intracellular Ca2+ stores (ER or SR). SOCE is thus necessary to allow the entry of Ca2+ ions to replenish depleted ER/SR and initiate intracellular Ca2+ signals [1]. In 2005, STIM1 was identified as a member of SOCE that located in ER and translocated into puncta to induce extracellular Ca2+ influx [2], followed by the identification of Orai1 [3] and transient receptor potential channel of the canonical family (TRPC); interestingly, TRPC1 contributes to SOCE is not direct but requires the interplay between STIM1 and Orai1 [4–8]. While TRPC3, TRPC6, and TRPC7 mediate SOCE, it is still debated [4, 9, 10]. Previous studies have found that upon Ca2+ store depletion, stromal interaction molecule1 gets accumulated at the endoplasmic reticulum-plasma membrane (ER-PM) junction to interact with Orai1 leading to the activation of Ca2+ ion release-activated Ca^2+^ channels [11–14]. According to the genetic research highlights, the significance of SOC on human health is well explained. These studies have shown that patients lacking or having mutations in stromal interaction molecule1, or Orai1 may suffer from severe health problems, including immune deficiency, autoimmunity, bleeding disorders, skeletal muscle disease (i.e. tubular aggregate myopathy, Stormorken disease, and York-platelets disease), and cardiac diseases [15–18]. SOC serves to generate Ca2+ ion signals that are delivered to gene transcription and subsequently, if any phenotypic changes occur, leads to cardiovascular pathology [19, 20]. This review provides a broad overview of the literature showing the role of Ca2+ influx via SOCE in cardiovascular diseases.

Endothelial dysfunction is the initiating factor of cardiovascular disease. As a non-excitable cell, there are two types of Ca2+ channels, L-type channels (Cav1.2) and SOC channels which are the majority; the rare is Cav1.2. A previous study found that Orai1-mediated Ca^2+^ influx decreased endothelial nitric oxide synthase (eNOS) expression induced by tunicamycin, which attributes to endothelial dysfunction [21]. This is consistent with our finding that Orai1 and TRPC1 regulated Ca^2+^ entry contribute to endothelial apoptosis [22]. SOC is also expressed in vascular smooth muscle cells (VSMC), mediating the receptor-activated Ca2+ pathway [23]. TRPC1-based SOCE contributes to contractility, proliferation, and migration and is a potential therapeutic target for cardiovascular diseases in VSMC [24–26]. However, evidence showed that Orai1 is independent of TRPC1-based SOC [23]. In contrast, using immunoprecipitation experiments, the passively depleted store with thapsigargin enhances TRPC1 association with Orai1 and STIM1 with Orai1 in VSMCs of aortic rats [27]. Moreover, data indicate for the first-time functional crosstalk between Orai1, TRPC1, and CaV1.2 channels, determining that upon agonist stimulation, vessel contraction involves Ca^2+^ entry due to co-localize of Orai1 and TRPC1 with CaV1.2 [28]. It is well known that phenotypic transformation of VSMCs is associated with cardiovascular disease, including hypertension and atherosclerosis [29]. The SOC has been reported to mediate the phenotypic switching of VSMC, which attribute to the activation of Ca2+/calmodulin-dependent protein (CaMKII)δ [30]. However, Ziomek et al. found that tunicamycin induced a significant increase of intracellular Ca^2+^ in VSMC. Still, it was independent of the activation of Ca2+ channels instead of the direct permeability of the plasma membrane, ER, and sarcoplasmic reticulum to Ca^2+^ [31]. The different results may be due to other cell types.

A consensus is that beat-to-beat Ca^2+^ handling is regulated by excitation–contraction coupling (ECC). Canonical ECC in cardiomyocytes is a Ca^2+^-induced Ca^2+^ release phenomenon. The process mainly relies on a combination of Ca^2+^ channel and transporters, more importantly, their precise locations and spatial arrangement. Although early evidence has revealed SOCE in adult myocytes [32, 33], store-operated Ca2+ entry in C57BL/6 J mouse ventricular myocytes and its suppression by sevoflurane is also reported. SOCE is a relatively new phenomenon and also controversial in cardiac myocytes. It is generally accepted that SOCE is more evident in the developing heart [34]. A common function of SOCE is to replenish depleted sarcoplasmic reticulum (SR) Ca^2+^ stores [35]. It is first identified that SOCE microdomains located in catecholaminergic ventricular tachycardia myocytes contribute to arrhythmogenesis through Ca^2+^ “spillover” from the SOC-mediated Ca^2+^ entry pool to the ECC pool. Key SOCE constituents, STIM1 and Orai1, participated in the process [36]. The absence of structural heart disease characterizes arrhythmia, implying that SOCE enhancement is insufficient for hypertrophic remodeling. However, the role of other constituents, including TRPCs, must await further studies. A previous study found that over-expression of STIM1 exhibited sudden death and heart failure. The death may be due to arrhythmia, while heart failure develops with increased cardiac hypertrophy and reduced cardiac function. The result may be associated with partially co-localize with type 2 ryanodine receptor (RyR2) and dysregulation of type Ca2+ channel (LTCC), which contributed to the enhancement of Ca2+ cycling in SR [37], the former distinct from in adult mouse skeletal muscle, which showed complete co-localization of STIM1 with RyR1 through the entire myofifiber [38]. It is worth saying that accumulation of STIM1 and Orai1 have been found in muscles tubular aggregates of aged mice [39]. Still, it is difficult to determine whether tubular aggregates form due to altered Ca2+ handling or if they are the cause of muscle dysfunction.

Moreover, calsequestrin-1, a protein that acts as the main Ca2+ buffer in the SR and plays a central role in skeletal ECC, has been proposed to mediate SOCE by a retrograde signal that inhibits STIM1 aggregation. The role of calsequestrin-1 in cardiomyocytes and arrhythmia may deserve further study. There are no specific tools to define the contribution of SOCE constituents. Thus, the role of the contribution of these proteins in arrhythmogenesis must await further studies. The specific component of SOC involved in the above process needs to be studied in depth.

## Pathological roles of store-operated channels in cardiovascular disease

### Arterial thrombosis

At the site of vascular injury, loss of endothelial cells exposes the subendothelial extracellular matrix; soon, platelets are quickly adhered and activated and form a closed lesion embolism with the coagulation system. This process is essential to prevent excessive blood loss, but in pathological situations, it may result in arterial thrombosis that may play a critical role in myocardial infarction and stroke [40]. Myocardial infarction elicited by coronary atherosclerotic plaque corrosion or rupture is one of the two major diseases causing disability and mortality worldwide [41]. Human platelets contain significant levels of Orai1, Orai2, and Orai3 and different transient receptor potential ion channel subfamily members [42, 43]. Unlike a mouse, human platelets have been described as a significant inherent distinction between platelet count levels and the expression of specific Ca2+ ion signals. However, these differences do not exclude employing the mouse models to clarify the Ca2+ ion pathway on platelets. SOCE is the predominant mechanism of intracellular Ca2+ signals [44]. In STIM1-deficient platelets, a severe deficiency occurring in the Ca2+ ion response to all major agonists confirmed that SOC acts as the main pathway for Ca2+ ion entry in platelets, which is crucial for downstream pathway glycoprotein (GP) Ib-GPVI-immunoreceptor tyrosine-based activation motif [45]. However, platelets lack the normal endoplasmic reticulum, and the intriguing problem is that the exact location of STIM1 and the specific molecules involved in regulation is still unclear. Probably, but to a minor extent, STIM2 may be a candidate molecule that has been proven to initiate store-operated channels [46].

Moreover, it was reported that the critical role of Orai1 and TRPC6 in platelets is induced by G protein-coupled receptor activation and thrombosis [47, 48]. From arterial thrombosis of the mouse model, it was shown that cyclophilin A (CyPA) was identified as a Ca2+ ion modulator in platelets for the first time. CyPA deficiency is severely inhibited by activation-induced Ca2+ ion fluctuation in the cell and extracellular Ca2+ ion influx, which strongly impaired platelet activation. This study determined that CyPA in regulating Ca2+ ions was a key mechanism for arterial thrombosis [49]. Moreover, Karen Wolf and his colleagues concluded that secreted platelet serotonin 5-hydroxytryptamine (5-HT) was necessary to enhance the second stage of platelet activation through store-operated channel-mediated Ca2+ influx, as it played a significant role in thrombus stabilization, which is mainly dependent on 5-HT receptor 2A mediated phospholipase C β signaling and amplified Orai1 activity [50]. Consistent with these results [51], it is essential to elucidate the mechanisms of signaling pathways that require further research. Recently, S.K. Gotru et al. [52] found that thapsigargin-induced activation of the STIM1 function resulted in a significant reduction of store-operated Ca^2+^ entry in transient receptor potential melastatin 7 (TRPM7)-deficient platelets. The results suggested a functional interaction between STIM1 and TRPM7, which contains a cytoplasmic domain of serine/threonine alpha-kinase [52]. A significant observation was the function of Orai1, and STIM1 was regulated by multiple phosphorylations of serine residues [53, 54]. Hence, further research is needed to investigate whether TRPM7 kinase can phosphorylate these residues in the store-operated Ca^2+^ entry protein complex. Therefore, it can be considered that regulatory proteins are involved in store-operated Ca^2+^ entry, which could be the target for antithrombotic therapy.

### Atherosclerosis

The complex interaction of modified lipoproteins and immune cells with arterial wall cellular components causes a chronic inflammatory process that promotes the formation and development of atherosclerosis [55–57]. For a long time, the formation of foam cells was considered an essential step in developing atherosclerosis. Recently it was demonstrated that knockdown of Orai1 with transfection small interfering RNA (siRNA) or SOCE inhibitor with SKF96365 dramatically inhibits atherosclerotic plaque development involved in macrophage scavenger receptors. This process is mainly dependent on calcineurin(CaN)-apoptosis signal-regulating kinase1 and its downstream effectors, c-Jun N-terminal kinase (JNK) and p38 mitogen-activated protein kinase, but not on the nuclear factor of activated T4 [58]. Unexpectedly, Orai1-mediated Ca2+ entry via the CaN-nuclear aspect of activated T cells (NFATc) 4 signals is a key inflammatory pathway required for endothelial cell activation and vascular inflammation induced by tumor necrosis factor-α (TNFα) [59]. The critical role of Orai1 in regulating endothelial and macrophages has been confirmed in-vivo and in-vitro; the different target receptors might be related to cell type.

Moreover, TRPC3 shows its impact on endothelial dysfunction and is responsible for causing apoptosis of macrophages related to the pathogenesis of atherosclerosis. The relevance of TRPC3 with atherosclerosis has also been verified by in vivo studies [60], where the process is associated with endoplasmic reticulum stress that is considered the primary mechanism of cell apoptosis in atherosclerotic plaques [61]. It is well known that endothelial barrier function dysfunction is regarded as a leading cause of atherosclerotic plaque formation.

Interestingly, Stolwijk et al. found that STIM1 may regulate endothelial barrier functions independent of store-operated Ca^2+^ entry [62]. In contrast, knockdown or inhibition of STIM1 controlled the high-mobility group box 1 protein-induced Ca2+ entry followed by significantly reduced endothelial permeability [63]. However, the difference was correlated with other different agonists and pathological processes.

Endothelial progenitor cells (EPC) were considered the source of vascular repair [64]. Previous studies have shown that store-operated Ca^2+^ entry is a crucial regulator of EPC function [65, 66]. Wang LY et al. in his study found that endothelial progenitor cells proliferation and migration activities were significantly reduced in atherosclerotic mice, as well as store-operated Ca^2+^ entry amplitude, that could be due to the Ca2+ ion oscillations related to the reduced expression of Orai1, STIM1, and TRPC in these cells [67]. Moreover, early growth response protein-1 has been shown in atherosclerosis of animal models and response to growth stimuli in VSMC [68]. The data demonstrated that angiotensin II-induced early growth response protein-1 mediated the STIM1/Orai1/Ca^2+^-dependent pathway. The role of STIM1, Orai1, and TRPC1 regulating Ca2+ influx in the atherosclerosis model has not been elucidated clearly. Hence, SOC-mediated Ca2+ ion entry may be a promising therapeutic target for atherosclerosis.

### Cardiac hypertrophy

Cardiac hypertrophy is the primary mechanism by which the heart responds to overloads such as myocardial infarction or hypertension to maintain pump function [17]. Calcium ion is an essential intracellular signal for cardiac hypertrophy to various stimulations [69]. The downstream signaling induced by Ca2+ ions initiates transcription procedures associated with cardiac hypertrophy, pathological growth, and cardiac remodeling, finally changing cardiac function. However, several studies have suggested that store-operated Ca^2+^ entry plays a key role in cardiac hypertrophy by altering the fetal genetic program controlled by the CaN/NFAT signals. In another study, Hulot et al. found that neonatal cardiomyocytes overexpress STIM1 and are significantly larger and showed an enhanced NFAT activity, which can be prevented in the presence of SKF-96365 [70]. Also, both STIM1 and Orai1 knockdown have entirely inhibited the growth of hypertrophic neonatal cardiomyocytes, which are mediated by phenylephrine and inhibited calmodulin kinase II and extracellular signal-regulated kinase 1/2 signaling, Orai1 deficiency occasionally prevented CaN-dependent hypertrophic pathway [71]. These data were obtained from in-vitro studies, which confirmed that store-operated Ca2+ entry is essential for developing pathological cardiac hypertrophy. However, its role in in-vivo models has not been studied.

From previous studies, it is understood that gastrodin weakened the activity of store-operated Ca^2+^ entry by reducing the expression of two essential proteins, i.e., STIM1 and Orai1, in vivo and in vitro. The data suggested that inhibition of store-operated Ca^2+^ entry can reduce myocardial hypertrophy induced by phenylephrine, which indicates that the STIM1-Orai1-Ca^2+^ dependent pathway is located upstream of hypertrophy [72]. However, the SOC regulator involved in the process has not yet been fully elucidated. Store-manipulated Ca^2+^ entry-associated regulatory factor (SARAF) is an intrinsic regulator of store-manipulated Ca^2+^ entry, facilitating the weakening of STIM1-Orai1 interaction [35, 73]. Dai F et al. showed that store-manipulated Ca2+ entry-associated regulatory factor overexpression suppressed STIM1 /Orai1 upregulation and cardiac hypertrophy induced by angiotensin II [74]. It is still unclear which specific mechanism of store-manipulated Ca^2+^ entry-associated regulatory factor regulates STIM1-Orai1; for example, store-manipulated Ca^2+^ entry-associated regulatory factor prevents STIM1 activation or affects the translocation of STIM1 from the endoplasmic reticulum to the plasma membrane. Also, TRPC is an essential mediator responsible for causing pathological myocardial hypertrophy. Moreover, it has been reported that TRPC1/5 dysregulation contributed to myocardial hypertrophy [75, 76]. However, in their study, the role of SOC in regulating TRPC1/5 has been reported to be unclear. A previous study found that inhibition of TRPC in transgenic mice or cultured neonatal cardiomyocytes remarkably reduced the activity of CaN-NFAT, which is a known Ca^2+^-dependent hypertrophy induction pathway. Therefore, TRPC contributes to the development of myocardial hypertrophy, partially through the CaN-NFAT signaling pathway [77]. Tang L and his coworkers recently established a stable human-based cardiomyocyte hypertrophy model and highlighted molecular mechanisms underlying TRPC1-mediated hypertrophy, which was related to abnormal activation of nuclear factor kappa-light-chain-enhancer of activated B cells (NF-κB) [78]. Understanding the mechanism of STIM1, Orai1 and TRPC1 may prevent cardiac hypertrophy or heart failure.

### Hypertension

Calcium ion is a central component that controls vascular contraction. It has been proposed that the abnormal treatment of cations in vascular myocytes may contribute to the response of vascular smooth muscle cells (VSMC) in contraction and stimulation that is enhanced by myogenic tension enhancement, which is a crucial marker of hypertension. Fernanda R.C reported that compared to age-matched male Wistar-Kyoto rats, blockade of SOC by 2-aminoethoxy diphenyl borate (2-APB) remarkably suppressed contraction in stroke-prone spontaneously hypertensive (SHRSP) rat aortas during the Ca^2+^ loading period [79]. This accordingly enhanced the activation of STIM1/Orai1 in the aorta of male SHRSP, which represents a mechanism that leads to impaired control of sex-related intracellular Ca2+ ion levels. In addition to this study, they even investigated female ovariectomy to understand whether it affects the activation of the Orai1/STIM1 pathway. The data suggested that female sex hormones may negatively regulate the STIM1/Orai1 pathway and contribute to vascular protection in female rats [80]. In a study conducted on smooth muscle-specific knockout of STIM1 of mice (stromal interaction molecule1 SMC^−/−^mice), Kassan et al. found that STIM1 was pivotal for causing hypertension. They demonstrated that after angiotensin II infusion at the 4^th^ week, STIM1 expression in the cardiovascular system of wild-type mice was enhanced, which was found to be associated with hypertension and endothelial dysfunction; however, hypertension was induced by angiotensin II, that significantly reduced in stromal interaction molecule1 SMC^−/−^mice [81]. Recently, it has been shown that low Ca2+ ion buffering capacity of partly depolarized mitochondria caused capacitative Ca^2+^ entry disorder, resulting in excessive endoplasmic reticulum- Ca2+ ion store by enhancing STIM1/Orai1 interaction and increased aorta contraction of spontaneously hypertensive rats (SHRs) [82]. Although several molecules participated in hypertension, the specific molecule of SOC-mediated Ca2+ entry in this pathogenesis is undefined. TRPC has been reported to increase in models of hypertension [83–85], especially a higher TRPC3 and change in TRPC 3/6 proportions in essential in hypertension, which is associated with depolarization of vascular smooth muscle cells [86]. Liu D et al. found that TRPC3 was significant in controlling Ca2+ ion entry in VSMC [87]. Previous research showed that TRPC3 transcription is closely related to systolic blood pressure ascribed to pro-inflammatory cytokines interleukin-1β (IL-1β) and tumor necrosis factor-α in essential hypertension [88].

Moreover, elevated TRPC3 messenger RNA (mRNA) levels in patients with hypertension were associated with increased salt intake and systolic blood pressure [89] but, its specific mechanism needs further investigation. The earlier studies were conducted in cultured rat and human embryonic kidney 293 cells; Parker et al. demonstrated that inhibition of sphingosine kinase 1 (SK1) attenuated the second phase of transmembrane Ca2+ ion influx in Ang II-mediated hypertension, suggesting a role for sphingosine kinase 1 in Ang II-dependent activation of SOC [90]. However, different experimental conditions, including specific vascular bed studies and other vasoactive drugs and animal species, led to heterogeneity results. Considering the effect of different vasoactive agents (such as Ang-II, Noradrenaline) on blood pressure, multiple receptor blockers are used to treat hypertension [91, 92]. Pharmaceutical experiments using SKF-96465 suggested that inhibition of store-operated Ca^2+^ entry positively affected blood pressure reduction and Ca2+ ion release induced by Ang-II [93]. Moreover, the SOC activity was reduced by tyrosine kinase inhibitors, which control blood pressure [94]. Accordingly, it would be of great significance to investigate the effects of SOC inhibitors in hypertension.

### Pulmonary arterial hypertension

The imbalance of pulmonary systolic and vasodilation is the cause of pulmonary hypertension. Importantly, pulmonary arterial remodeling is a pathological alteration. Mechanistically, it is involved in the proliferation of pulmonary artery smooth muscle cells (PASMC). Cytosolic free Ca^2+^ [(Ca^2+^)i] plays a pivotal role in pulmonary vascular remodeling [95]. Furthermore, the alteration in Ca2+ ion signaling and profound pulmonary vascular remodeling may lead to pulmonary arterial hypertension (PAH) [96]. Accumulating evidence has implicated that store-operated Ca^2+^ entry is responsible for developing pulmonary arterial hypertension [97–99]. Wang J et al. suggested that Orai1/2/3 and STIM1 contributed to SOC-mediated Ca^2+^ influx in PASMCs, especially Orai2 is found to be a hypoxia-inducible factor-1α(HIF-1α)-dependent [98]. Following this, Fernandez et al. demonstrated that Orai2 knockdown reduced store-operated Ca^2+^ entry in proliferative PASMCs. The authors further found that proliferative pulmonary artery smooth muscle cells have enhanced store-operated Ca^2+^ entry. Moreover, STIM2 was implicated in increased activity of store-operated Ca^2+^ entry [100]. Interestingly, an increase of STIM2 in PASMC was observed in patients with idiopathic pulmonary hypertension. The role of STIM2 in store-operated Ca^2+^ entry may attribute to its effect on the augmentation of PASMC proliferation. However, STIM1 was not significantly changed in idiopathic pulmonary hypertension-PASMC [101]. Previously, STIM1 knockdown reduced store-operated Ca^2+^ entry and decreased hypoxia-induced PASMC proliferation, suggesting that STIM1 significantly impacts hypoxic pulmonary arterial hypertension [102], and CaN/NFAT shows increased activity in pulmonary arterial hypertension. Moreover, the CaN/NFAT signaling pathway has been involved in pulmonary artery smooth muscle cell proliferation through monocrotaline-induced pulmonary arterial hypertension [103]. Recently, Dong F et al. found that Chrysin inhibited the hypoxia-induced promotion of pulmonary artery smooth muscle cells proliferation and store-operated Ca^2+^ entry, which may be associated with inhibition of TRPC1 and TRPC6 [104]. Similarly, in vitro experiments suggested that the TRPC antagonist SKF-96365 controlled pulmonary artery smooth muscle cells proliferation and decreased expression of TRPC1, TRPC6, andCaN/NFATc3 caused by 5-HT [105]. The correlation between 5-HT and TRPC may promote the pathogenesis of pulmonary arterial hypertension. Previous studies confirmed that store-operated channels play a critical role in pulmonary arterial hypertension. However, different induced conditions can result in different pathogenesis and pathological manifestations. Notably, the models cannot accurately mimic human pulmonary arterial hypertension. The STIM, Orai, and TRPC in pulmonary arterial hypertension patients need further investigation. Future studies aim to understand the role of these proteins in pulmonary arterial hypertension patients and the irregulatory pathways.

**Fig. 1:**
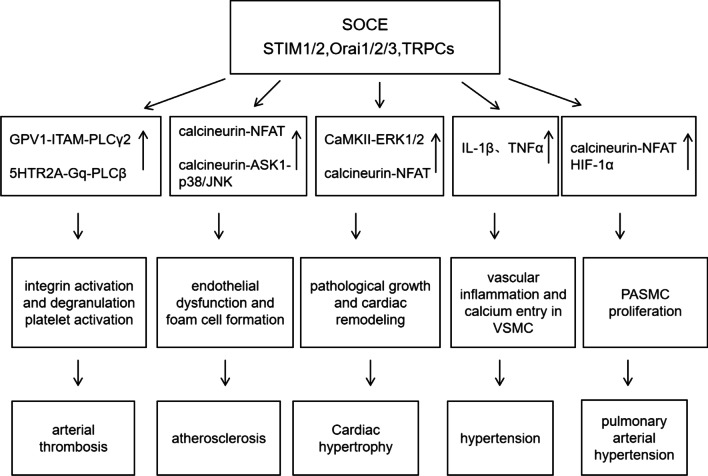
Illustration of involvement of abnormal SOCE in cardiovascular diseases and possible underlying mechanisms. Upper arrows show activation of pathway. GPV1: glycoprotein V1; ITAM: immunoreceptor tyrosine-based activation motif; 5HTR2A: 5-hydroxytryptamine receptor 2A; PLCβ: phospholipase C beta; NFAT: nuclear factor of activated T cell; ASK1: apoptosis signal-regulating kinase 1; CaMKII: calmodulin kinase II; ERK1/2: extracellular signal-regulated kinase 1/2; IL-1β: interleukin-1beta; TNF-α: tumor necrosis factor-alpha; VSMC: vascular smooth muscle cell; HIF-1α: hypoxia inducible factor 1α; EPC: endothelial progenitor cells; PASMC: pulmonary artery smooth muscle cell

The role of store-operated channels in cardiovascular diseases has been summarized in Fig. 1.

## Conclusion

Accumulating evidence indicates that approaches aimed at store-operated Ca2+ entry in the cardiovascular system may be therapeutic. The pharmacological tools have demonstrated the contribution of SOCE constituents in cardiovascular diseases; while useful for initial investigation and analysis, they lack specificity. Although the constituents of SOC were shown to play a central role in Ca2+ signals, their absolute requirement for store-operated Ca2+ entry regulation is helpful to carry out further investigation. The Cav1.2 and colocation with SOC have been reported for the first time in vessel contraction. The effect of functional crosstalk in hypertension and atherosclerosis might be worthy of further study. Besides, Ca2+ originating from SOCE could modulate cell adhesion and ECC by influencing intercalated disk-residing proteins' functions. The role of SOCE in maintaining the intercalated disk structure and function via regulation of protein synthesis, trafficking, and targeting may be a future research direction of arrhythmia. There is a hope that future studies will reveal further signaling molecules regulating cardiovascular Orai1/STIM1 and TRPC abundance and its function. Evaluation of SOC channels will shed new light on the treatment of cardiovascular disease.

## Data Availability

The raw data supporting the conclusions of this article will be made available by the authors without undue reservation.

## Abbreviations

SOCs: Store-operated channels
ER: Endoplasmic reticulum
SR: Sarcoplasmic reticulum
InsP3: Inositol 1,4,5-trisphosphate
STIM1: Stromal interaction molecule 1
Ca2+: Calcium ions
Orai1: Ca2+ release activated Ca2+ channel protein 1
PM: Plasma membrane
CRACs: Ca2+ ion release-activated Ca2+ channels
TRPC: Transient receptor potential channel of canonical family
Cav1.2: L-type channels
VSMC: Vascular smooth muscle cells
eNOS: Endothelial nitric oxide synthase
CaMKII: Ca2+/calmodulin-dependent protein
ECC: Excitation-contraction coupling
SR: Sarcoplasmic reticulum
GP: GGlycoprotein
CyPA: CyclophilinA
5-HT: 5-Hydroxytryptamine
TRPM7: Transient receptor potential melastatin 7
siRNA: Small interfer RNA
CaN: Calcineurin
JNK: C-Jun N-terminal kinase
NFATc: Nuclear aspect of activated T cells
TNFα: Tumor necrosis factor-α
EPC: Endothelial progenitor cell
SARAF: Associated regulatory factor
NF-κB: Nuclear factor kappa-light-chain-enhancer of activated B cells
2-APB: 2-Aminoethoxy diphenyl borate
SHRSP: Stroke-prone spontaneously hypertensive
IL-1β: Interleukin-1β
mRNA: Messenger RNA
SK1: Sphingosine kinase 1
PASMC: Pulmonary artery smooth muscle cells
PAH: Pulmonary arterial hypertension
HIF-1α: Hypoxia-inducible factor-1α
